# Girls just wanna have funds: a new Transparent Reporting Scale for evaluating grant data reporting from funding agencies

**DOI:** 10.3389/fncom.2026.1765249

**Published:** 2026-02-13

**Authors:** Natasha Clarke, Abigail E. Licata, Soumaiya Imarraine, Thuy Dao, Ginevra Sperandio, Ana Luísa Pinho, Valentina Borghesani, Paola Mengotti, Antonietta Gabriella Liuzzi, Doris Pischedda

**Affiliations:** 1 Department of Psychology, University of Montreal, Montreal, QC, Canada; 2 Research Centre of the Institut Universitaire de Gériatrie de Montréal, Montreal, QC, Canada; 3 Department of Psychology, University of Geneva, Geneva, Switzerland; 4 Neurosciences Paris Seine-Institut de Biologie Paris Seine (NPS-IBPS), INSERM, CNRS, Sorbonne Université, Paris, France; 5 Laboratoire Jean Perrin-Institut de Biologie Paris Seine (LJP-IBPS), CNRS, Sorbonne Université, Paris, France; 6 School of Information Technology and Electrical Engineering, University of Queensland, St Lucia, QLD, Australia; 7 Institute of Medical Psychology and Medical Sociology, University Medical Center Schleswig-Holstein, Kiel University, Kiel, Germany; 8 INSERM U987. APHP, CHU Ambroise Paré, UVSQ, Paris-Saclay, Boulogne-Billancourt, France; 9 Department of Psychology, Western Centre for Brain and Mind, Western University, London, ON, Canada; 10 Department of Computer Science, Western University, London, ON, Canada; 11 Cognitive Neuroscience, Institute of Neuroscience & Medicine (INM-3), Forschungszentrum Jülich, Jülich, Germany; 12 Laboratory for Cognitive Neurology, Department of Neurosciences, Leuven Brain Institute, KU Leuven, Leuven, Belgium; 13 Department of Brain and Behavioral Sciences, University of Pavia, Pavia, Italy

**Keywords:** DEI (diversity, equity, and inclusion), gender equity, grants, neuroscience, open science, research funding, science policy, STEM

## Abstract

**Introduction:**

Despite the increasing representation of women in scientific fields, disparities in research funding allocation remain. This inequity deprives talented women researchers of necessary resources, limiting the diversity of perspectives and ideas, and contributes to the “scissor-shaped curve” seen in neuroscience, where women leave before obtaining senior positions. Data transparency and comprehensive reporting of information on grant winners and applicants, as well as reporting of gender and other intersecting demographics and key metrics, are crucial to effectively evaluate funding equity. However, there is a lack of guidelines on which data funders should report. In this study, we aimed to investigate the transparency of neuroscience funders across Europe, focusing on the European Union, Schengen area, and the United Kingdom.

**Methods:**

To this end, we developed a Transparent Reporting Scale (TRS), composed of 15 items crucial to facilitate transparent and meaningful reporting, and searched for public data from funders in order to apply the scale and evaluate their transparency in data reporting. Across 32 countries and the European Union as a whole, we identified 39 funders, with 90% sharing publicly available data on funding results.

**Results:**

Using the TRS, five funders received a “gold” rating, eighteen a “silver” one, and thirteen a “bronze” rating. Scale scores were significantly correlated with the Gender Equality Index [*p* = 0.64, 95% CI (0.33, 0.83), *p* = 0.001] and gross domestic product of the countries where funders are based [*p* = 0.51, 95% CI (0.20, 0.74), *p* = 0.003], suggesting that collection and/or publication of funding data may reflect overall commitments to gender equity, and be limited due to resources. Data from only 29% of funders could be disaggregated for the neuroscience category specifically, indicating the difficulty in evaluating equity in our field.

**Discussion:**

We collated all available data into an Open Science Framework repository to enable data sharing and further analyses. The TRS can support funders in adopting transparent, standardized reporting practices in order to support evidence-based progress toward gender equity.

## Highlights

- We analyzed gender and diversity data reporting from 39 European neuroscience funders.
- The TRS was developed to benchmark funders’ commitment to data transparency.
- Five funders achieved a “gold” rating, demonstrating that comprehensive and accessible reporting is feasible.
- Almost 60% of funders reported data disaggregated by gender, and 57% reported data for all applicants, not just winners, which is crucial to evaluate success rates.
- TRS scores were significantly correlated with the Gender Equality Index and gross domestic product of the countries where funders are based.
- The TRS can support funders in improving transparency, accountability, and equity in neuroscience research.

## Introduction

Despite commendable strides from the scientific community in addressing gender inequity in research and academia (Dworkin et al., 2020; Fulvio et al., 2021; Llorens et al., 2021; Joyce et al., 2024), women remain underrepresented in STEM (science, technology, engineering, and maths), comprising less than 30% of researchers globally (Lewis et al., 2021). In the European Union (EU), where gender equality in research and innovation is a priority, women hold only 20% of top academic positions despite representing 37% of doctoral STEM graduates (European Commission: Directorate-General for Research and Innovation, 2025). This loss of women’s talent with career progression – termed the “leaky pipeline” – stems from complex systemic factors, including gender bias in grant review and funding decisions that negatively impact women’s academic careers (Sato et al., 2021; Bourke et al., 2023; Joyce et al., 2024). Funding is often explicitly used as a criterion for promotion (Schmaling and Gallo, 2023) and is also key in validating scientific ideas, enabling research, and supporting publication, all of which are critical determinants of academic career success.

Research findings confirm gender bias in funding across countries, funders, and career stages (Boyle et al., 2015; van der Lee and Ellemers, 2015; Burns et al., 2019), with multiple factors that may disadvantage women. Analysis of publications suggests that women’s work is undervalued: they receive fewer citations than men (Dworkin et al., 2020; Chatterjee and Werner, 2021; Lerman et al., 2022) and are less likely to be named on collaborative work (Ross et al., 2022). Men early in their careers also cite themselves more than their women counterparts (Rosenblatt et al., 2025), and publish more at higher levels of seniority (European Commission: Directorate-General for Research and Innovation, 2025), factors which may be linked. Gender stereotypes may also distort grant evaluations, with implicit biases about scientists evident from a young age (Asplund and Welle, 2018). The language of grant applications may also bias funding: instructions and evaluation materials have been found to use gendered language favoring male applicants (van der Lee and Ellemers, 2015), and analysis of data from the Canadian Institutes of Health Research found that women were rated less favorably than men only when the focus of the evaluation was on leadership, as opposed to the proposed project (Witteman et al., 2019). Finally, women remain underrepresented in leadership roles critical to shaping the funding landscape. In the EU, around 39% of board members, and 26% of institution leaders, are women (European Commission: Directorate-General for Research and Innovation, 2025), and men make up a large percentage of neuroscience and psychology journal editors (Palser et al., 2022).

Fewer women also apply and reapply for grants than the number eligible to make applications (Boyle et al., 2015; Hechtman et al., 2018; Schmaling and Gallo, 2023). While the reasons for this are not clear, there is evidence that women spend a disproportionate amount of time on diversity, equity, and inclusion (DEI) work (O’Meara et al., 2017) and teaching activities (Gibney, 2017), which likely impacts the time they spend on grant writing. Caregiving responsibilities, which disproportionately impact women (Cech and Blair-Loy, 2019; Morgan et al., 2021), may also have a negative impact. This was particularly evidenced during the COVID-19 pandemic, when a disparity in childcare and caregiving led to worse outcomes for women, including research productivity (Manchester et al., 2023; McLachlan, 2023; Thébaud et al., 2024) and specifically the rate of grant submissions (Berge et al., 2023). Women also receive smaller awards and fewer awards upon reapplication (Schmaling and Gallo, 2023).

Furthermore, the impact of other individual characteristics, and their intersection with gender, may also impact equitable funding. One study found that while men’s success rate remains stable with age, women’s success declines after age 50 (Boyle et al., 2015). At the United Kingdom Research & Innovation Agency (UKRI), Principal Investigators (PIs) who reported a disability were less likely to be funded, and received lower funding amounts, than those who did not report a disability, and twice as many grants were held by male PIs with a disability than women PIs with a disability. Only 1% of PI awardees were black, and 57% of awardees were white men (UK Research and Innovation [UKRI], 2023). Race was also found to intersect with gender in receiving and applying for National Institutes of Health (NIH) awards in the United States (Ginther et al., 2018), and in negative outcomes of the COVID-19 pandemic (Berge et al., 2023). These findings illustrate how intersecting barriers contribute to a cumulative disadvantage for women and other underrepresented groups, and suggest a complex impact of gender on funding, which may contribute to the leaky pipeline.

However, some findings are encouraging. A large meta-analysis of 55 peer-reviewed grants from Europe and North America between 1975 and 2020 found no significant gender differences in funding success rates (Schmaling and Gallo, 2023), although data was not stratified by field, which may dilute gender differences (Boyle et al., 2015). For example, in the United Kingdom (UK) fewer women apply to biomedical funding agencies, and men receive grants that are 15% larger, yet no such differences are seen in the social sciences, where the proportion of women is similar (Boyle et al., 2015). In an effort to improve the status quo, the Irish Research Council (2020) introduced a gender-blind grant assessment process, which resulted in a substantial increase in women being awarded the STEM postdoctoral program, from 35% in 2013 to 57% in 2017. Along the same line, measures to combat gender bias from the Dutch Research Council, such as proportional allocation of funding to men and women, prioritization of applications from women in ex aequo situations, implicit bias training to panelists, more inclusive wording of instructions for panelists and reviewers, and transparent reporting of the amount of women applicants and awardees, led to more women being awarded grants than men (Albers et al., 2024).

Neuroscience poses particular challenges in evaluating and addressing gender disparities. It is rarely confined to a single department, instead spanning biology, psychology, medicine and other disciplines. This structural ambiguity means neuroscience is often overlooked in large-scale evaluations of gender bias, and is not always clearly captured within traditional STEM policy frameworks designed to promote equity in science. Despite attracting a relatively high proportion of women at early career stages, only around 29% of authors of neuroscience papers are women (Hakvoort et al., 2023), and representation declines sharply at senior levels (Araneda-Guirriman et al., 2023; Bourke et al., 2023; Spoon et al., 2023). This disparity between men and women researchers at senior levels, termed the “scissor-shaped curve” (Joyce et al., 2024), is likely exacerbated by the field’s internal stratification: women tend to come from subfields such as cognitive, developmental, and affective neuroscience, which are often perceived as less prestigious and “softer” than male-dominated areas like systems or computational neuroscience (Fulvio et al., 2021; Morgan et al., 2021). These patterns reflect not only systemic gender bias but also the need for a more nuanced, field-specific approach to understanding and addressing inequities in research funding. The neuroscientific community has also made particular headway in conducting open and reproducible science, guided by principles such as FAIR (data being Findable, Accessible, Interoperable, and Reusable, Wilkinson et al., 2016).

Although some institutions and funding agencies have adopted new strategies to support gender equity in funding acquisition, there is a lack of standardized guidelines on how gender and other individual characteristics should be collected and reported (Ruediger et al., 2025). Existing reporting is highly inconsistent, often incomplete, and rarely disaggregated by gender or other intersecting demographics. This opacity prevents applicants, institutions, and policymakers from evaluating equity, identifying structural barriers, and monitoring progress. Full transparency from funders of both application and success rates by gender and other individual characteristics is essential, but reporting bias and missing data are common (Schmaling and Gallo, 2023). Public access to this information enables greater insight for potential applicants and better tracking of progress in eliminating biases, ensures that funding bodies can be held accountable, and offers opportunities to learn from more equitable fields.

Inspired by open science practices, we aimed to collect public data on neuroscience funding application and award rates, disaggregated by gender and other individual characteristics, with a view to conducting an analysis of the field. It became apparent that reporting practices varied drastically across funders, thus, we developed the Transparent Reporting Scale (TRS) in order to evaluate funders of neuroscience grant programs, to promote and aid in the reporting and monitoring of grantee demographics. In this paper, we focus on members of the EU, Schengen area and the United Kingdom. By analyzing freely available data and reports, each funder was scored based on whether they reported disaggregated data on competition applications and results, in-line with the FAIR principles. Available data was collated, and is made available in an Open Science Framework (OSF) repository, as a first step to improving transparency and further analysis of potential biases in STEM funding. We also make available an online tool to score the TRS^1^, to encourage re-evaluation.

## Materials and methods

### Data collection and extraction

We conducted an online search for large-scale funding institutions (herein referred to as “funders”) across the EU-27 member states, Schengen area and the United Kingdom, that offer neuroscience research grants. Data was initially collected between 2020 and 2025, and all data was double-checked between September and December 2025. We started our search from public national funding agencies for each country (e.g., Deutsche Forschungsgemeinschaft-DFG, for Germany). In case we did not know the national funder of a country, we asked colleagues/collaborators from that country. We looked for any data reporting funding awarded over any time period, on the funders website or using Google search engine. Keywords in the search included, but were not limited to, “Neuroscience,” “Funding,” “Diversity,” and “Report.” Our goal was not to perform a comprehensive search but to identify at least one funder per country. We included all national, public funders as well as large private or umbrella funders widely used in the neuroscience field (e.g., the European Research Council-ERC). Where possible, we focused on data specific to neuroscience funding. If neuroscience-specific funding results were not available, we looked for and summarized data provided as a whole. We documented and recorded links to all data found, and data that was available to download was added to a publicly available OSF repository^2^. From each data source found, we extracted the data type (report, list, tabular or available in a portal) and the years covered by the reporting. We then rated each data source according to the TRS, outlined below.

### The Transparent Reporting Scale (TRS)

We developed the TRS, a new scoring tool to evaluate funders’ ability to assess and transparently report gender and other characteristics, inspired by FAIR principles (Wilkinson et al., 2016). To build the scale, we established 15 meaningful criteria, grouped into five categories (see below). These criteria enable analysis, interpretation, and tracking of potential biases in funding applications and awards.

A: Accessibility and FAIR principles

1. Findable: Were we able to find publicly available data via the funding agency or programs website, or using a search engine?
2. Downloadable: Can data be downloaded? This ensures accessibility and supports reproducible and transparent research.
3. Tabular format: Is data organized in a table, such as a spreadsheet? This enables interoperability of data and further analysis.
4. English: Is the data provided in English?

B: Gender/DEI and demographics

1. Gender: Is data about the gender of the applicant/winner reported?
2. Age or career stage: Is the (biological/academic) age of the applicant/winner or their career stage reported? These are combined into a single sub-point since both of these metrics could indicate a similar bias in terms of age or career stage (for example, if younger women or women in junior positions receive less funding).
3. Parental leave: Does the funder have publicly available information on parental leave policies, such as what happens to the funding in the case of parental leave, for either gender? This may be particularly important for individuals considering grant applications who are planning to become parents.
4. DEI page: Does the funder have publicly available information on policies or a commitment to DEI in evaluating and providing funding?

C: Temporal information

1. Year of award: Is there information about when the grant was awarded?
2. Grant duration: Is the duration of the grant reported?
3. Longitudinal: Is data reported for three or more years? Can trends be assessed? This demonstrates a commitment to transparency and is crucial for analyzing any changes over time.

D: Funding

1. Summary funding: Is the total amount of funding awarded reported?
2. Individual funding: Is the amount given to each awardee reported?
3. All applicants (not just winners): Is data reported for *all* applicants or only for grant winners? Reporting of data for all applicants is important in order to assess equity in funding allocation according to the number of applicants, and assess who is applying for grants, as opposed to only those who are successful.

E: Field

1. Neuro-specific: All funders considered include neuroscience projects, but often embedded in macro-categories, such as medicine, psychology, and biology. Is it possible to search for results of neuroscience-specific funding? Data need to include (or could be disaggregated based on) a specific ‘neuroscience’ category or ERC-based panel structure (LS5-Neuroscience and Disorders of the Nervous System, and SH4-The Human Mind and its Complexity).

Each criterion can score 1 or 0. Scores for each point in the scale are summed up with equal weight. Therefore, the maximum score on the TRS is 15. If a funder had data available in multiple formats (e.g., a report on their website and downloadable tabular data), they were first rated separately, and in further analysis, we took their highest possible score. Therefore, scores reflect the entirety of the data published by the funder rather than any specific format, since lower-scoring data sources would provide less information. Based on the total score, each funder received a transparency rating: bronze (1–8 points), silver (9–12 points), or gold (13–15 points). We also noted whether ethnicity or disability was reported; however, these criteria were not included in the scale due to differing rules across countries for collecting such sensitive data.

### Scoring process

The scoring was performed by 5 of the authors (Natasha Clarke, Abigail E. Licata, Paola Mengotti, Antonietta Gabriella Liuzzi, Doris Pischedda), independently. We adopted an objective scoring method, therefore, each author scored different funders and it was not necessary to calculate inter-rater reliability. For each scale item, we assigned a score of 1 if the answer to the relative question (see previous paragraph) was positive and 0 otherwise. For example, for the “downloadable” criterion, if the answer to the question “Can data be downloaded?” was positive, we assigned a score of 1. In case of doubt, the item was scored by at least another person, until agreement was reached.

### Relationship between reporting and socio-economic indices

We tested whether there was an association between TRS scores and the (a) Gender Equality Index (GEI), which measures progress in gender equality in different domains across EU countries, developed by the European Institute for Gender Equality (2025), and (b) Gross Domestic Product (GDP) per capita, by calculating the Spearman’s rank correlation coefficient. Confidence intervals (CI) for Spearman’s *ρ* were estimated via Fisher z-transformation. We obtained the most recent data available: GEI data for 2025 were obtained from the European Institute for Gender Equality website^3^, and GDP data for 2024 were obtained from the World Bank using the world-bank-data Python library. For both analyses, for countries with multiple funders, we took only the maximum TRS score, to highlight what is possible in reporting as opposed to penalizing lower TRS scores, however we also tested the correlations using the minimum and mean TRS scores per country.

### Data visualization and analysis

Data was visualized using Python. All code is available at https://github.com/WomenInNeuroscience/funders_project. We report descriptive statistics for the TRS and its subcategories. We also provide code to easily calculate scores on the TRS, using Python.

## Results

**Figure 1:**
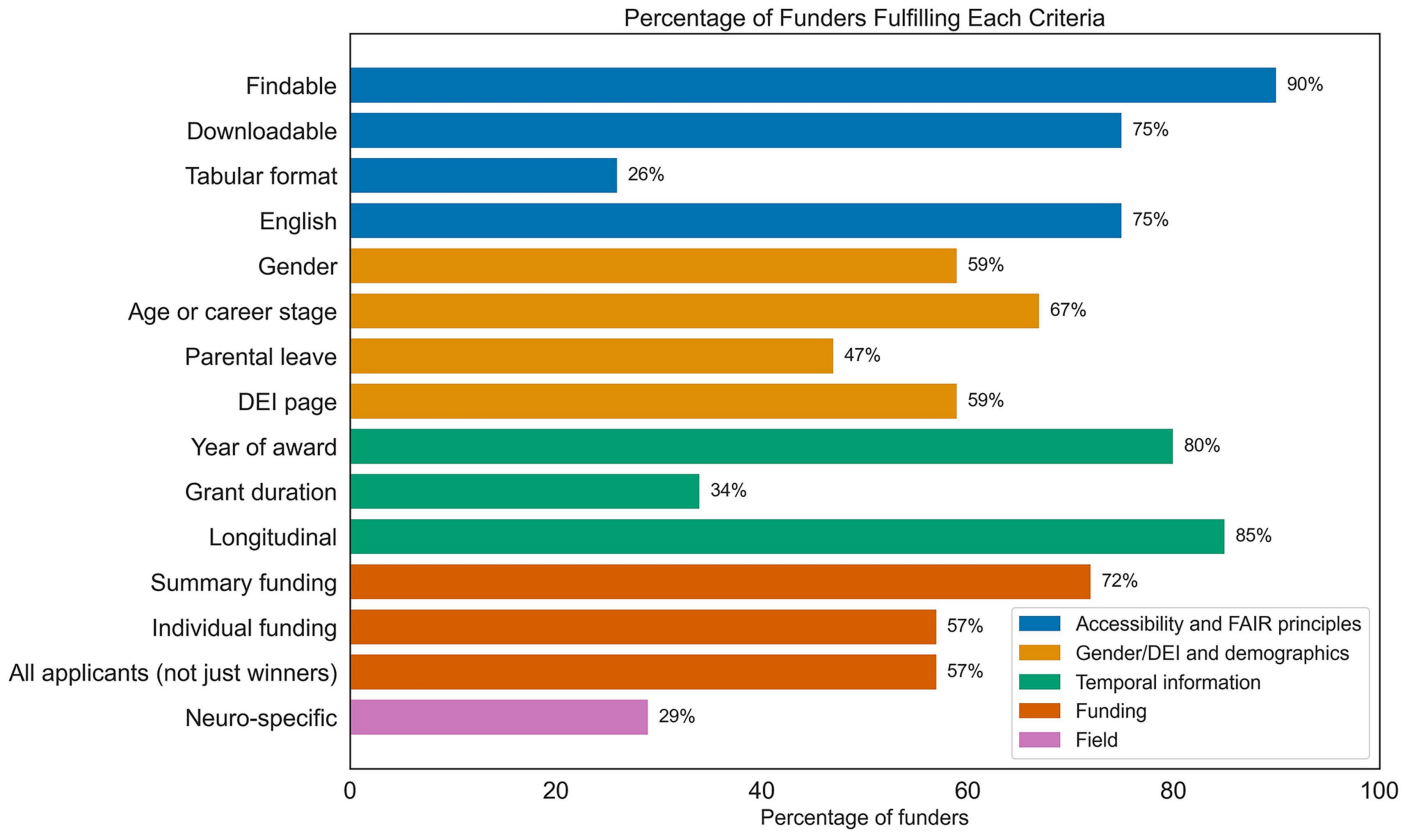
Percentage of funders fulfilling each criterion of the Transparent Reporting Scale, colored according to the different domains.

The percentage of funders fulfilling each criterion of the TRS is shown in Figure 1, and we report the results per category below.

### Accessibility and FAIR principles

Across 32 countries and the EU as a whole, we identified 39 funders that offer neuroscience research grants. Of these, 90% (35/39) shared publicly available data on funding results, and 75% (29/39) shared downloadable data. Only 26% (10/39) shared data in tabular format. The majority of funders (75%, 29/39) shared data in English. Across all 53 data sources, the majority was provided in an annual or one-off report (47%, 25/53).

### Gender/DEI and demographics

Examining DEI metrics, 59% reported data on gender (23/39), and 67% (26/39) reported age or career stage (65% (25/39) reported career stage while 26% (10/39) reported age). Information on parental leave could be found for 47% (18/39) of funders, while 59% (23/39) had a webpage or information on their DEI policies. Although not part of the TRS scoring, we note that 8% (3/39) of funders reported ethnicity or disability.

### Temporal information

**Figure 2:**
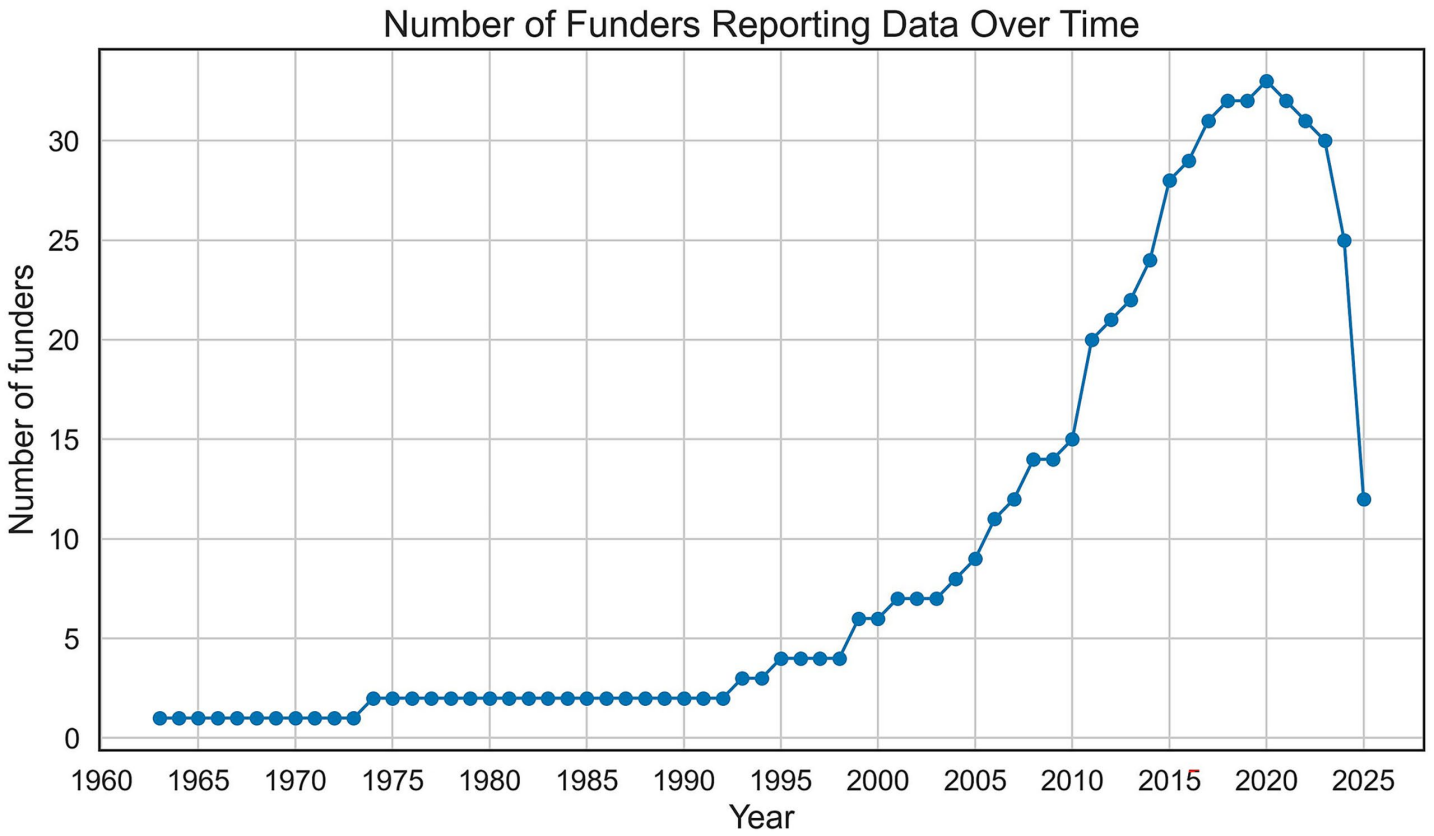
The number of funders reporting any data over time.

The year of award was reported by 80% (31/39) of funders, while 34% (13/39) reported the grant duration and 85% (33/39) published data over three or more years. The earliest data available was from 1963 (Novo Nordisk Foundation, Denmark). After 1993, the number of funders reporting data steadily increased (see Figure 2) until 2020, after which a drop in reporting in the recent years (2021–2025) was observed.

### Funding

The total amount of funding awarded was reported by 72% (28/39) of funders, while 57% (22/39) reported the amount given per individual awardee. Data for all applicants, as opposed to only those who were successful, was provided by 57% (22/39) of funders.

### Field

Neuroscience-specific data was retrievable or could be disaggregated in a minority of funders (29%; 11/39), whereas in the majority of cases neuroscience projects were embedded into macro-categories (e.g., medicine, psychology, biology).

### TRS scores

**Figure 3:**
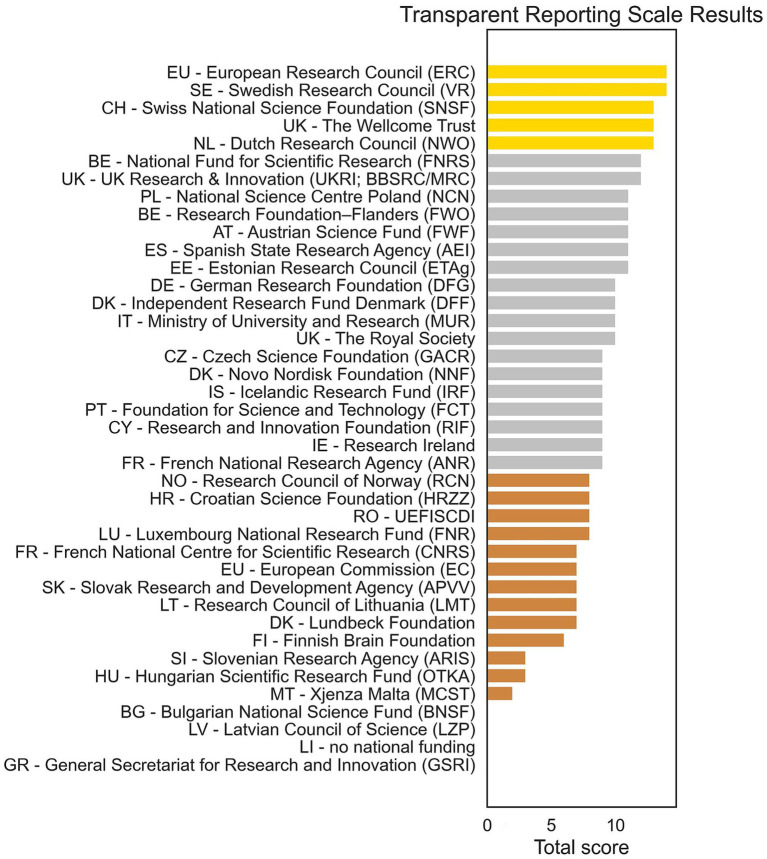
Transparent Reporting Scale scores for each funder. Colors refer to the gold, silver, and bronze categories from the transparency rating.

We applied the TRS to the 35 funders with available data (see Figure 3). A gold rating was awarded to five funders: the ERC, Swedish Research Council, Swiss National Science Foundation, The Wellcome Trust (United Kingdom) and the Dutch Research Council. Silver was awarded to 18 funders, and bronze to 13 funders. No data could be found for three funders, receiving a rating of zero, and one country (Liechtenstein) had no national funding programs. A full breakdown of TRS scores is available as a .csv file at both the GitHub repository^4^ and the OSF project page (see Footnote 2).

### Criteria fulfilled by funders

**Figure 4:**
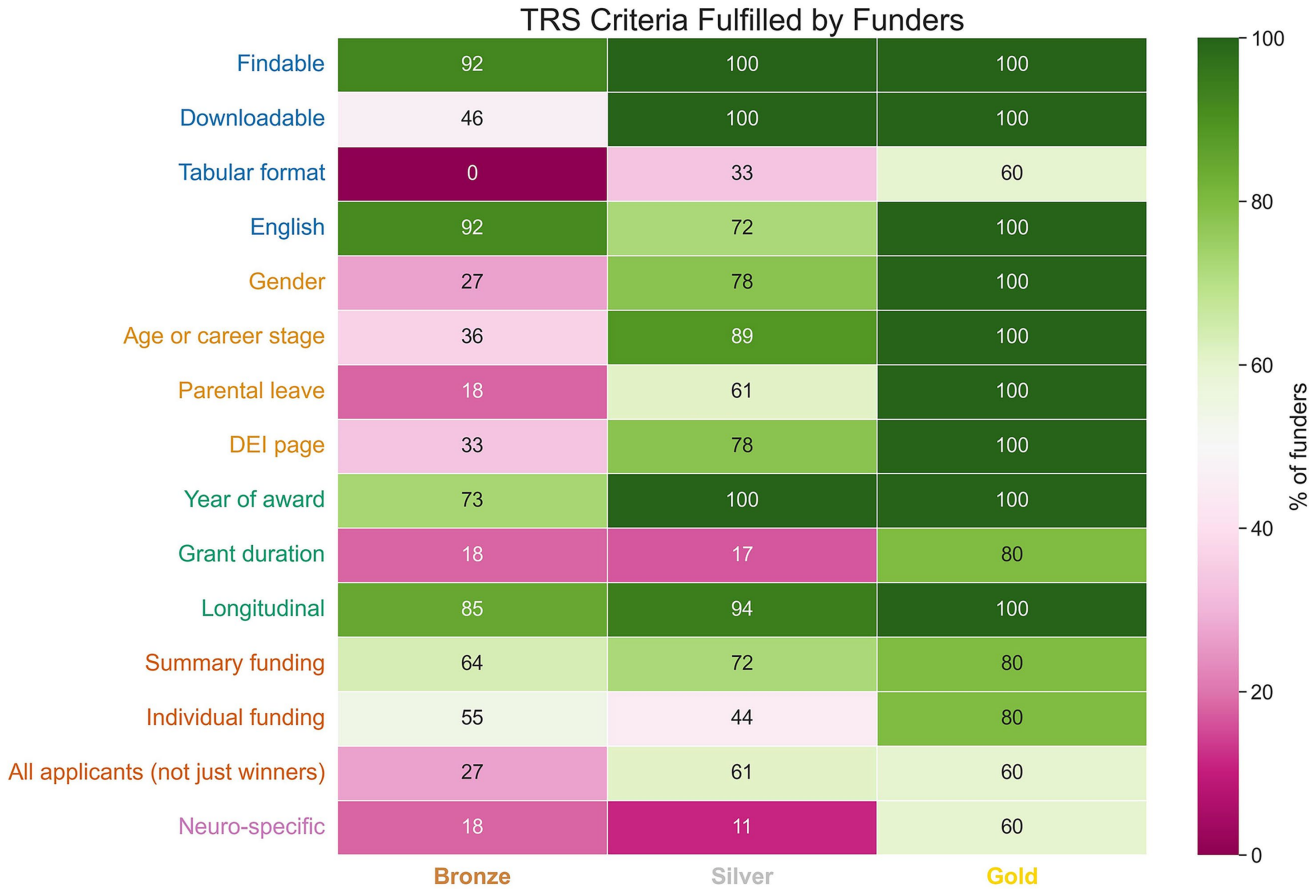
Percentage of funders achieving a bronze, silver, or gold TRS score fulfilling each criteria. Each criterion is grouped and colored corresponding to the five TRS domains (matching Figure 1).

The percentage of funders in the bronze, silver and gold scoring categories fulfilling each TRS criteria can be seen in Figure 4. As with other reported results, this analysis takes the highest score awarded to a funder if multiple data sources were found. Gold funders met all criteria of the “Gender/DEI and demographics” domain (in yellow), and most of the “Accessibility and FAIR principles” domain (in blue), with the exception of publishing tabular data. Four criteria were well-met across bronze, silver and gold funders: having findable data, reporting in English, reporting the year of an award, and reporting data longitudinally (over at least 3 years).

### Relationship between reporting and socio-economic indices

**Figure 5:**
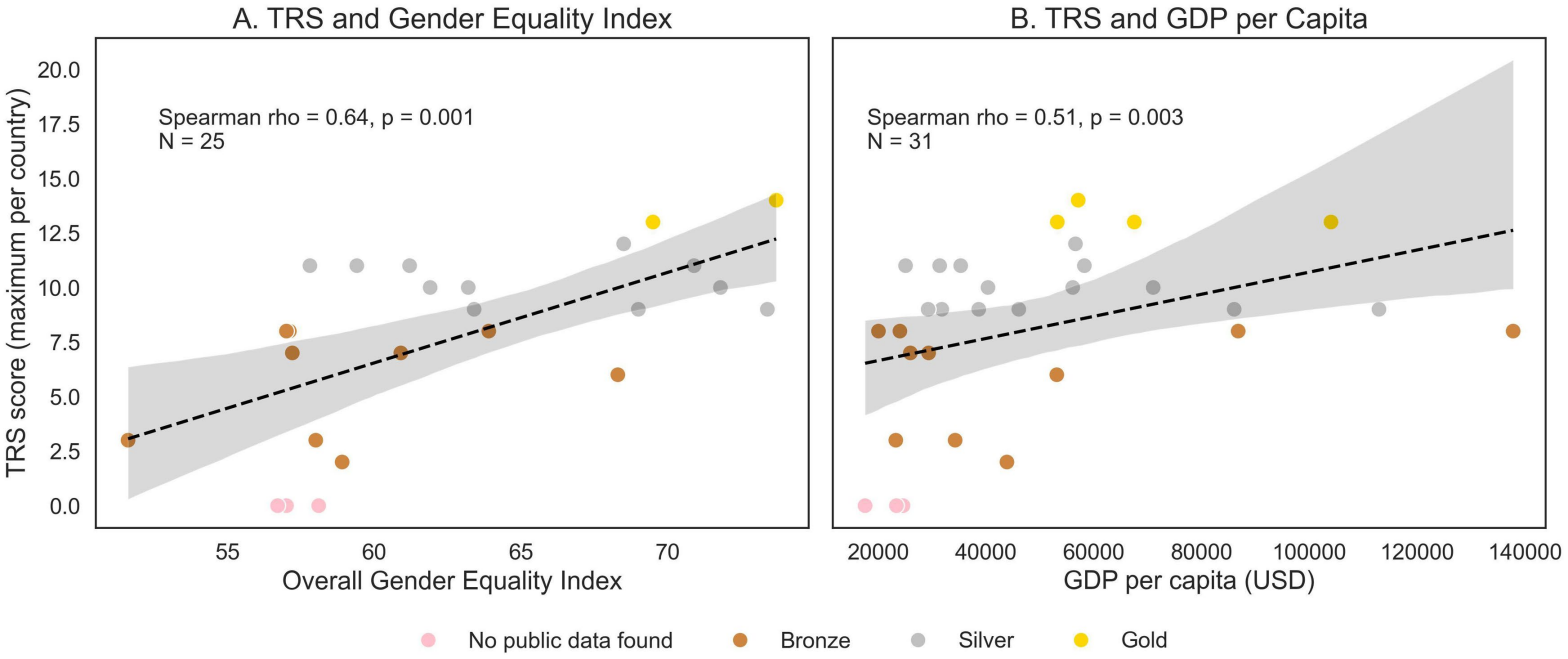
Association between TRS scores and **(A)** Overall Gender Equality Index for each EU country, and **(B)** GDP per capita for all countries with available data.

Since Liechtenstein did not have a national funder, and therefore had no data to report, it was excluded from correlation analyses. EU-wide funders were also excluded, since their funding schemes are open to the United Kingdom and Schengen area countries, and therefore do not match EU-level GEI and GDP indicators. Spearman’s rank correlation was used to examine the association between TRS scores and the GEI for each country in the EU with available data (*N* = 25), and each country’s GDP (*N* = 31), as the data were non-normally distributed. There was a significant positive correlation between TRS scores and both the GEI [*ρ* = 0.64, 95% CI (0.33, 0.83), *p* = 0.001] and GDP per capita [*ρ* = 0.51, 95% CI (0.20, 0.74), *p* = 0.003] of the countries where funders are based, indicating that funders from wealthier countries with higher GEI scores tended to score higher on the TRS (Figure 5). Results using minimum TRS score, and mean TRS score, per country, were similar for both GEI [minimum TRS: *ρ* = 0.52, 95% CI (0.162, 0.761), *p* = 0.007; mean TRS: *ρ* = 0.605, 95% CI (0.28, 0.81), *p* = 0.001] and GDP [minimum TRS: *ρ* = 0.454, 95% CI (0.118, 0.696), *p* = 0.010; mean TRS: *ρ* = 0.50, 95% CI (0.17, 0.72), *p* = 0.004] correlations.

### Data availability and storage

The OSF repository contains 43 data records from 29 funders, spanning 25 countries or the whole of Europe, from 1974 to 2025. It is publicly accessible at https://osf.io/9cy6a/, and organized according to the format “Country-Funder.”

## Discussion

### The Transparent Reporting Scale

We aimed to assess the transparency of European funders (EU member states, Schengen area, and the United Kingdom) in sharing demographic and other data important for the evaluation of equitable research funding, motivated by the FAIR principles (Wilkinson et al., 2016). To meet this goal, we developed the TRS, offering a structured, reproducible framework for assessing reporting practices and rated funders, acting as a benchmark. By aggregating publicly available funding data into a single repository, we provide researchers with an unprecedented opportunity to evaluate and compare potential biases and progress over time. We aimed to evaluate transparency in neuroscience funding; however, only data from 29% of funders could be disaggregated specifically for the neuroscience category. Others included neuroscience data pooled with other fields, for example falling under Life Sciences, Health or Psychology. This lack of specific data limits the conclusions that can be drawn about transparency and equity in funding within neuroscience and supports the notion that neuroscience poses a particular challenge in evaluating and addressing gender disparities (Fulvio et al., 2021; Morgan et al., 2021).

Overall, five funders received a “gold” TRS rating, which provide funding across Europe or in Sweden, Switzerland, the United Kingdom or the Netherlands, demonstrating that comprehensive and accessible reporting is feasible. The predominance of “silver” ratings indicates widespread efforts to be transparent with funding data. Our rating should not be taken as discouraging or as punishing funders who showed a less structured, efficient or complete sharing of their data, but rather show a way forward, by following the practices of the funders with the higher ratings. However, the lack of findable data from a small number of funders suggests that structural barriers to monitoring equity in research funding persist, consistent with evidence that gender and other biases remain challenging to track systematically (Boyle et al., 2015; van der Lee and Ellemers, 2015; Schmaling and Gallo, 2023). We found a correlation between transparency and a country’s GDP, suggesting that data collection and/or sharing may be partially explained by financial or organizational resources, though this correlation should not be interpreted as causal. We also observed a correlation between transparency and the GEI, which measures a country’s progress in gender equality. While also only an association, this result provides evidence of convergent validity for the TRS, as it suggests that transparency in funding practices co-occurs with broader structural commitments to equity.

Our analysis revealed that the availability of data has improved over time, with a rise in public reporting since the 1990s (see Figure 2), however with a decrease in reporting from 2020 on. We can only speculate on the reasons behind this decrease. Missing reporting in the most recent years (2020–2023) involve mostly funders who publish the data in the form of an annual report. The delay may be due to a delay in writing such reports, as data are often presented in a processed form. Other reasons may include the fact that not all programs are offered annually, or the impact of COVID-19, if grants were not provided or funders did not have the infrastructure to publish data. Missing data from 2023 to 2025 may simply reflect delays in data sharing. The majority of funders shared some data, often in English and downloadable, supporting findability and accessibility of data. However, the scope, quality and structure varied substantially. Data was provided in different formats, with the majority embedded in annual or one-off reports, limiting interoperability (Wilkinson et al., 2016). Almost 60% of funders reported data on all applicants (not only awardees) and provided the amount awarded per grantee. These data are essential for assessing potential biases, as prior work shows that women apply for funding less often and, when successful, tend to receive smaller awards than men (Boyle et al., 2015; Hechtman et al., 2018; Schmaling and Gallo, 2023). These metrics are also critical for evaluating the effects of COVID-19, which disproportionately affected women in the workplace, including in the rate of grant submissions (Berge et al., 2023).

Gender remains the most widely studied source of bias in science (Lewis et al., 2021; European Commission: Directorate-General for Research and Innovation, 2025). A number of factors, including citation practices (Dworkin et al., 2020; Rosenblatt et al., 2025), teaching loads (Gibney, 2017; O’Meara et al., 2017), lower representation in senior and decision-making positions (Palser et al., 2022; Joyce et al., 2024; European Commission: Directorate-General for Research and Innovation, 2025), and birthing or adopting a child disproportionately impact women in STEM careers compared to men (Cech and Blair-Loy, 2019; Morgan et al., 2021). A particularly strong form of gender bias is the maternity bias, which affects women with children or who are pregnant. In fact, mothers are less likely to be hired or to get a promotion (Wolfinger et al., 2008), they receive lower salaries and fewer job offers (Kelly and Grant, 2012), and are regarded as less competent than fathers and people without children (Correll et al., 2014). These factors can accumulate across women’s careers and create a more challenging environment, making them less favorable recipients of funding (Sato et al., 2021). However, intersectional factors such as age, race, ethnicity and disability, can compound barriers to funding (Berge et al., 2023; Boyle et al., 2015; Ginther et al., 2018; UK Research and Innovation [UKRI], 2023). The reporting of career stage can serve as a useful proxy for age-related inequities, as research shows that women in later career stages or older age receive less funding than men at equivalent stages (Boyle et al., 2015). Our TRS results for DEI reporting revealed a lack of transparency across funders: 67% reported age or career stage, and 59% reported gender. While not included in the TRS specifically, only 8% reported ethnicity or disability. This limited reporting hinders the identification and remediation of potential biases, particularly intersecting ones, in funding allocation.

The underreporting of DEI data may reflect limited awareness of its importance, resource constraints for data collection and analysis (supported by the finding of an association with GDP), reluctance to disclose potentially unflattering findings, or national regulations that restrict the collection of sensitive data. However, the consequences of this lack of transparency are significant, as it perpetuates a system in which biases can persist, limiting opportunities for underrepresented groups in neuroscience. In our data, very few funders reported ethnicity or disability, with United Kingdom funders being the exception. Although collection of such sensitive demographic data is generally permitted in EU member states, national laws may limit what is collected, with some countries focusing instead on nationality or citizenship. Socioeconomic status, a key factor in academic pathways (Morgan et al., 2022), may also impact funding equity, and intersect with gender and other demographics (Ruediger et al., 2025); however, to our knowledge, funders do not collect or publish this data. Additionally, only 44% of the funders mention parental leave in their grant guidelines, providing information about what would happen to the funding (e.g., grant time extension) during the leave time. Some funders, such as the ERC, show best practices to this regard, taking into account time dedicated to care for children in correcting academic seniority for eligibility, with an 18-month flat-rate extension per child.

### Recommendations to improve inclusivity in research funding

Some policies have already been proposed to improve inclusivity in the research funding system. Torres et al. (2024) discuss some of these measures aimed at promoting inclusion of caregivers (particularly women), that could help address gender bias (especially maternity bias) and inequalities in research funding. For example, extending grant eligibility criteria or track-record evaluation by at least 18 months per child would support continuity in research and reduce the impact of career gaps (e.g., missed grant calls, delayed manuscript submissions, lost data collection time). Moreover, providing the same paid parental leave options to both parents, could help mitigate gender disparity in workforce participation and parents’ income, and foster a more equal sharing of caregiving duties by mothers and fathers, while, at the same time, being more inclusive for same-gender couples. Funders could play an important role in this respect by providing additional paid paternity and maternity leaves to make parental leave opportunities more equitable. Funders should also provide flexible funding to allow for hiring support figures to perform lab work during parental leave, in order to ensure continuity in research activities. Additional measures could be implemented to reduce gender bias. For example, funders could mention actions to counter this bias in the reviewer guidelines, reporting clear examples and citing relevant laws against discrimination, or even provide specific training with proven effectiveness to reviewers.

Furthermore, Gender-Net Plus^5^, a European research project funded under the Horizon 2020 program, provides practical recommendations for funders to promote gender equality. Importantly, they highlight six concrete actions: (1) National authorities should support and monitor funding agencies’ efforts to promote gender equality. (2) Funding agencies should have a permanent structure (department or task force) to monitor a clear Gender Equality Plan. (3) Evaluation panels and decision-making bodies should aim for at least 40% representation of each gender. (4) Funding agencies should encourage women to apply, design calls inclusively and account for career breaks. (5) They should also make funding processes, evaluation criteria and feedback publicly available. Finally, and completely in line with our work on developing the TRS, they recommend that (6) funding agencies should collect, analyze and publish gender-disaggregated data on applicants, grantees and reviewers to access trends and gaps. This mirrors our approach and reinforces our view that funding agencies should record application and award data comprehensively and in a systematic way, and make it easily accessible to the public to improve transparency in resource allocation. Recording data from all applicants (including gender, etc.) is particularly important, as gender imbalance is often present already at this stage. This would also allow further analysis to identify the main sources of biases and the most effective measures to prevent them.

### Limitations

A limitation of this work is that our analysis was constrained by the authors’ geographical and language knowledge, which may introduce bias. Where possible, data in other languages was evaluated if an author spoke the language or it could be translated online; however, this was not always feasible (for example, data embedded in files that online translation tools could not recognize). The TRS criterion of reporting in English is also biased, but necessary, as a common language used across scientific publishing that enables comparisons across countries, and benefits researchers seeking funding in other countries. We also acknowledge that our list of funders is not exhaustive, and other agencies may demonstrate good practices in transparent reporting that are not captured here. The goal of this paper is to present the TRS and demonstrate its feasibility with preliminary data; future work should expand its use to other countries to provide a more comprehensive view of funding transparency and potential biases worldwide. We also note that high transparency does not imply a lack of bias, but is rather a critical step for evaluation and insight. Finally, we recognize that gender is non-binary. Current reporting typically uses a binary classification (women/men); identifying non-binary individuals can pose a risk to privacy, but we hope that future work will enable more gender inclusive analyses of funding equity.

## Conclusion

This work provides a systematic analysis of transparency in data sharing among European (EU, Schengen area, and the United Kingdom) funders, and introduces a new tool to serve as an evidence-based benchmark. We envision the TRS being adopted by funders not just to aid in retrospective analysis of reporting, but also as a tool to follow when collecting and analyzing data, adjusting accordingly to ensure transparency and comprehensive DEI reporting practices. The collation of available European data in the OSF is a first step to further analysis, and we hope that this effort can be built upon by the research community, also extending data and findings to other countries. By making this information readily available, we can better track progress toward equitable funding and the impact of policies, hold funders accountable, and identify areas where interventions are needed. Beyond transparency, we recommend implementing policies to actively address potential biases in the review process, such as unconscious bias training for reviewers, standardized evaluation criteria, and greater diversity among review panels (see Schrouff et al., 2019 for a detailed discussion of these issues). While our study focused on European funding agencies and programs, the findings have broader relevance for the global research community, across neuroscience and other STEM fields. A collective effort to prioritize DEI in research funding is essential to ensure that talented scientists from all backgrounds can fully contribute to the advancement of science.

## Data Availability

The datasets presented in this study can be found in online repositories. The names of the repository/repositories and accession number(s) can be found: https://github.com/WomenInNeuroscience/funders_project, https://osf.io/9cy6a/.

## Footnotes

1
https://www.winrepo.org/transparency_calculator

2
https://osf.io/9cy6a/

3
https://eige.europa.eu/gender-statistics/dgs/indicator/index__index_scores/datatable

4
https://github.com/WomenInNeuroscience/funders_project

5
https://gender-net-plus.eu
